# Supplementary material for: From sequence to enzyme mechanism using multi-label machine learning
Source: BMC Bioinformatics. 2014 May 19;15:150. doi: 10.1186/1471-2105-15-150 (PMC4229970; doi:10.1186/1471-2105-15-150)
Supplement: Additional file 2 — Java code of ml2db. Additional file ml2db_code.tar.gz contains the Java source code to run the multi-label machine learning experiments and save the results to database. The code’s Javadoc is included. [file 1471-2105-15-150-S2.zip › additional file 2/ml2db/utils/doc/uk/ac/ed/inf/utils/collectionutils/setutils/SupersetsManager.html]

SupersetsManager


---


|  |  |  |
| --- | --- | --- |
| **Overview** \| **Package** \| **Class** \| **Use** \| **Tree** \| **Deprecated** \| **Index** \| **Help** | |  |
| **PREV CLASS**   NEXT CLASS | **FRAMES**    **NO FRAMES**     **All Classes** |  |
| SUMMARY: NESTED \| FIELD \| CONSTR \| METHOD | DETAIL: FIELD \| CONSTR \| METHOD |  |


---


## uk.ac.ed.inf.utils.collectionutils.setutils Class SupersetsManager

```
java.lang.Object
  uk.ac.ed.inf.utils.collectionutils.setutils.SupersetsManager
```

---

``` public class SupersetsManager extends java.lang.Object ```

A class to identify the unique supersets among sets of interpro signatures. A
set is a subset of a given superset if all its elements belong to the
superset. For example: a-b, or a-c, or b-d are subsets of a-b-c-d, but a-e is
not. A set is always a subset of itself.
Beware not a very generic class (null are represented as '-1'), if -1 has a
meaning in your data, modify the class.

**Version:**
:   16 Mar 2011

**Author:**
:   Luna De Ferrari luna.deferrari-at-ed.ac.uk

---

| **Field Summary** | |
| --- | --- |
| `static boolean` | `m_debug` |


**Constructor Summary**

`SupersetsManager(java.lang.String sqlQuery,
DbManager dbManager,
java.lang.String separator)`

`SupersetsManager(java.util.TreeMap<java.lang.String,java.lang.String> setsIdsElements,
java.lang.String separator)`


| **Method Summary** | |
| --- | --- |
| `java.util.TreeSet<Set>` | `createSupersets()` |
| `void` | `generateSupersets()` |
| `java.lang.String` | `getSeparator()` |
| `java.util.TreeSet<Set>` | `getSets()` |
| `java.util.TreeSet<Set>` | `getSupersets()` |
| `java.lang.String` | `getSupersetsCSV()`             Get a comma separated list of [superset, subset] couples. |
| `OneToManyMap` | `getSupersetsIds()` |
| `java.lang.String` | `getSupersetsIdsCSV()`             Get a comma separated list of [superset id, subset id] couples. |
| `static void` | `main(java.lang.String[] args)` |

**Methods inherited from class java.lang.Object**

`equals, getClass, hashCode, notify, notifyAll, toString, wait, wait, wait`

**Field Detail**

### m\_debug

```
public static final boolean m_debug
```

**See Also:**: Constant Field Values


**Constructor Detail**

### SupersetsManager

```
public SupersetsManager(java.lang.String sqlQuery,
                        DbManager dbManager,
                        java.lang.String separator)
```

**Parameters:**: `sqlQuery` - to get the id + string of set elements divided by the separator: `dbConnectionPath` -: `separator` -

---


### SupersetsManager

```
public SupersetsManager(java.util.TreeMap<java.lang.String,java.lang.String> setsIdsElements,
                        java.lang.String separator)
```

**Parameters:**: `sqlQuery` - to get the id + string of set elements divided by the separator: `dbConnectionPath` -: `separator` -


**Method Detail**

### createSupersets

```
public java.util.TreeSet<Set> createSupersets()
```

---


### generateSupersets

```
public void generateSupersets()
```

---


### getSeparator

```
public java.lang.String getSeparator()
```

---


### getSets

```
public java.util.TreeSet<Set> getSets()
```

---


### getSupersets

```
public java.util.TreeSet<Set> getSupersets()
```

---


### getSupersetsCSV

```
public java.lang.String getSupersetsCSV()
```

:   Get a comma separated list of [superset, subset] couples.

    :   **Returns:**: a comma separated list of [superset, subset] couples.

---


### getSupersetsIds

```
public OneToManyMap getSupersetsIds()
```

---


### getSupersetsIdsCSV

```
public java.lang.String getSupersetsIdsCSV()
```

:   Get a comma separated list of [superset id, subset id] couples.

    :   **Returns:**: a comma separated list of [superset id, subset id] couples.

---


### main

```
public static void main(java.lang.String[] args)
```


---


|  |  |  |
| --- | --- | --- |
| **Overview** \| **Package** \| **Class** \| **Use** \| **Tree** \| **Deprecated** \| **Index** \| **Help** | |  |
| **PREV CLASS**   NEXT CLASS | **FRAMES**    **NO FRAMES**     **All Classes** |  |
| SUMMARY: NESTED \| FIELD \| CONSTR \| METHOD | DETAIL: FIELD \| CONSTR \| METHOD |  |


---
